# Extracting Knowledge from Machine Learning Models to Diagnose Breast Cancer

**DOI:** 10.3390/life15020211

**Published:** 2025-01-31

**Authors:** José Manuel Martínez-Ramírez, Cristobal Carmona, María Jesús Ramírez-Expósito, José Manuel Martínez-Martos

**Affiliations:** 1 Department of Computer Science, University of Jaén, E-23071 Jaén, Spain; jmmr0049@red.ujaen.es (J.M.M.-R.); ccarmona@ujaen.es (C.C.); 2 Andalusian Research Institute in Data Science and Computational Intelligence, DASCI, University of Jaén, E-23071 Jaén, Spain; 3 Leicester School of Pharmacy, DeMontfort University, Leicester LE1 7RH, UK; 4 Experimental and Clinical Physiopathology Research Group CVI-1039, Department of Health Sciences, University of Jaén, E-23071 Jaén, Spain; mramirez@ujaen.es

**Keywords:** breast cancer, serum biomarkers, explainable AI, oxytocin, early diagnosis, peptide hormones, IRAP, progesterone

## Abstract

This study explored the application of explainable machine learning models to enhance breast cancer diagnosis using serum biomarkers, contrary to many studies that focus on medical images and demographic data. The primary objective was to develop models that are not only accurate but also provide insights into the factors driving predictions, addressing the need for trustworthy AI in healthcare. Several classification models were evaluated, including OneR, JRIP, the FURIA, J48, the ADTree, and the Random Forest, all of which are known for their explainability. The dataset included a variety of biomarkers, such as electrolytes, metal ions, marker proteins, enzymes, lipid profiles, peptide hormones, steroid hormones, and hormone receptors. The Random Forest model achieved the highest accuracy at 99.401%, followed closely by JRIP, the FURIA, and the ADTree at 98.802%. OneR and J48 achieved 98.204% accuracy. Notably, the models identified oxytocin as a key predictive biomarker, with most models featuring it in their rules. Other significant parameters included GnRH, β-endorphin, vasopressin, IRAP, and APB, as well as factors like iron, cholinesterase, the total protein, progesterone, 5-nucleotidase, and the BMI, which are considered clinically relevant to breast cancer pathogenesis. This study discusses the roles of the identified parameters in cancer development, thus underscoring the potential of explainable machine learning models for enhancing early breast cancer diagnosis by focusing on explainability and the use of serum biomarkers.The combination of both can lead to improved early detection and personalized treatments, emphasizing the potential of these methods in clinical settings. The identified markers also provide additional research and therapeutic targets for breast cancer pathogenesis and a deep understanding of their interactions, advancing personalized approaches to breast cancer management.

## 1. Introduction

Breast cancer is a disease in which breast cells grow abnormally and uncontrollably [1,2,3]. These cells can form a tumor, which may become palpable as a lump [2]. Invasive ductal carcinoma is the most common type of breast cancer. It originates in the cells lining the ducts of the breast and spreads outside the duct into the surrounding breast tissue [1]. Approximately 80% of breast cancers are ductal carcinomas. Ductal carcinoma in situ (DCIS) is a non-invasive cancer that originated in the cells lining the ducts of the breast and has not spread outside the duct into the surrounding breast tissue [4]. Invasive lobular carcinoma originates in the lobules of the breast, where milk is produced, and spreads into the surrounding breast tissue [2,3].

The risk of breast cancer increases with age. Approximately 66.6% of breast cancer patients are older than 55 years [1,5,6,7,8]. Having a close relative with breast cancer, especially a first-degree relative, is also a risk factor [6,9]. Certain genetic mutations, such as mutations in the BRCA1 and BRCA2 genes, significantly increase the risk of breast cancer [8]. Prolonged exposure to estrogen, such as the early onset of menstruation, late menopause, or the use of hormone therapy, also increases the risk [6,8]. With regard to lifestyle, alcohol consumption, obesity, and a lack of physical activity are associated with an increased risk of the disease [2,6].

Prevention currently includes breast self-examination to detect any changes, such as lumps, skin changes, or nipple discharge. Screening and early detection methods include mammography [9,10,11], an X-ray of the breast used to detect breast cancer in the early stages, even before a lump can be felt, breast ultrasounds [8,11,12,13], which use sound waves to determine whether a lump in the breast is solid or a fluid-filled cyst, and breast MRI [8,13,14], an imaging test that uses a magnetic field and radio waves to create more detailed images of breast tissue and which is used to detect cancer in women at high risk or to better evaluate a finding from mammography or a physical examination. For definitive diagnosis, a biopsy is performed [5,10]. Ultimately, the treatment of breast cancer depends on several factors, such as the stage of the cancer, the type of cancer, and the age and general health of the patient. The treatment options include surgery, which may involve the removal of the tumor, part of the breast, or the whole breast (mastectomy) [5,8,10]; radiotherapy, which uses high-energy X-rays to destroy cancer cells [10,11]; chemotherapy, which uses drugs to destroy cancer cells [8,10,13,15]; hormone therapy, which is used to block the effects of hormones on cancer cells; and targeted therapy, which uses drugs that target specific characteristics of cancer cells [8,10,15].

Breast cancer is the most common cancer among women in the United States. It is estimated that there were about 2.9 million new cases in 2022 [7,16,17]. Globally, breast cancer accounts for 11.7% of cancer diagnoses and contributes significantly to mortality, especially among women over the age of 50 [1,8,18]. In 2020, there were 2.2 million new cases and 684,996 deaths from breast cancer [18]. The breast cancer incidence in 2020 was highest in Asia (45.4%) and Europe (23.5%), followed by North America (12.5%) and Latin America and the Caribbean (9.29%) [18]. Africa and Oceania reported lower incidence rates of 8.25% and 1.14%, respectively [7,18]. Asia also led in terms of breast cancer mortality with 50.5% of deaths, while Europe’s mortality rate stood at 20.7%, probably reflecting better healthcare outcomes [18]. Africa’s considerable mortality rate of 12.5% highlights critical challenges in medical care. In Spain, breast cancer causes 35,000 new cases per year and is the leading cause of cancer-related mortality in women, with 6651 deaths per year [6,18].

An early diagnosis of breast cancer is directly associated with a higher chance of survival [5,10,11,12]. This is because early detection allows for treatment to be initiated in a timely manner, which increases the chances of success and reduces the risk of the disease spreading to other parts of the body (metastasis) [1,5,11,12]. The treatment options are also broader and less aggressive [6,11]. Late diagnosis may imply the need for more invasive treatments, such as a mastectomy instead of breast-conserving surgery [5,9,10]. In addition, chemotherapy and radiotherapy, which can have considerable side effects, may be necessary at later stages [5,10,15]. In general, breast cancer treatment is most effective when initiated early in the disease because cancer cells are more sensitive when the tumor is small and has not spread [5,10,11,12]. The early diagnosis of breast cancer therefore allows patients to preserve a better quality of life as treatments are less invasive and have fewer side effects, allowing patients to maintain a more active and healthy life [11]. Finally, the early detection of breast cancer can reduce long-term healthcare costs because early-stage treatments are generally less expensive than treatments for advanced breast cancer [6,11].

In any case, it is important to emphasize the importance of early diagnosis in breast cancer management because it is crucial for improving survival rates and preventing breast cancer recurrence [5,6,10,11,12]. In fact, technology-based diagnostic tools are emerging. For example, a liquid biopsy is a non-invasive method for breast cancer screening that utilizes cancer-related biomolecules in the blood. Surface-enhanced Raman spectroscopy (SERS) combined with machine learning algorithms has shown great potential for rapid and accurate breast cancer detection. This technology enhances the Raman spectroscopy signal of serum using a specialized substrate, allowing for the discovery of new biomarkers. SERS combined with machine learning could achieve 100% accuracy, sensitivity, and specificity in distinguishing breast cancer patients from controls [19,20]. Similarly, Raman spectroscopy has been used with deep learning algorithms for screening triple-negative or HER-2-positive breast cancers [21].

Most data mining and machine learning studies mainly focus on the use of medical images and demographic data in studies of breast cancer diagnosis and prognosis [1,5,8,9,12,13,14,16,22,23,24]. However, serum biochemical parameters have been much less studied. Nevertheless, it is important to note that the integration of serum biomarkers can improve the accuracy of machine learning models for breast cancer risk prediction, early diagnosis, treatment response prediction, or recurrence detection [8,13,16,17]. Furthermore, the use of serum biomarkers offers a less invasive and more efficient approach for the early detection and monitoring of disease, also allowing for a binary classification of whether a patient has breast cancer or not. Unlike imaging techniques, which may have limitations in terms of their sensitivity and specificity, serum biomarkers can reflect early molecular changes associated with cancer development. In the context of the medical workflow, the extraction of serum biomarkers is performed through a blood test, a routine and minimally invasive procedure. This sample can be obtained at various stages of the healthcare process, from initial screening to monitoring the response to treatment. While conventional screening focuses primarily on mammography and other imaging techniques such as ultrasounds and MRI, it is important to note that these techniques have certain limitations. For example, mammography may not be as effective in women with dense breast tissue, and both ultrasounds and MRI can be expensive and are not available everywhere. In addition, these imaging techniques often detect cancer when a palpable tumor has already formed. Therefore, several artificial intelligence (AI) tools could be applied to a dataset of serum biomarkers with information on both patients and healthy individuals. By defining a classification problem [25], models could be trained in order to extract information from the previously mentioned dataset to diagnose whether a patient had breast cancer based exclusively on these biomarkers, which could prove useful to diagnose breast cancer when used along with other detection methods.

Though these tools often achieve highly accurate predictions, given the context of the problem, it is of the utmost importance that the results are not only precise but also explainable. The implemented systems should be able to explicitly define the reasons why their predictions occur, which in turn will allow the specialists who make use of them to discover new patterns in the data that may aid further research. For this purpose, black-box models, such as deep learning models, were ignored, and only models that offer rules or trees that grant information on specific markers that are relevant for the diagnosis of breast cancer were used in an effort to achieve trustworhy AI (TAI) [26].

## 2. Materials and Methods

### 2.1. Subjects

A total of 198 women were recruited in the Unit of Breast Pathology at the University Hospital of Jaén, and 78 volunteer women without breast cancer were also included as a control group. This study was approved by the Ethical Committee of the University Hospital of Jaén, and all subjects signed a declaration of free, informed consent. All women with breast cancer were diagnosed with ductal infiltrating carcinoma. A total of 83 of these women (39 premenopausal and 44 postmenopausal) did not receive neoadjuvant chemotherapy, whereas 115 of them (63 premenopausal and 52 postmenopausal) received neoadjuvant chemotherapy before surgery. The clinicopathological characteristics of the studied patients have been previously described [27,28,29,30,31]. The patient characterization included the age at diagnosis, tumor size, tumor histology, pathologic T classification, Scarff–Bloom–Richardson grade, hormonal and HER-2/neu status, and molecular subtype. The control group consisted of 78 women, aged 28 to 69 years old (premenopausal women with regular menstrual periods, n = 38; postmenopausal women with spontaneous menopause for at least one year, n = 40), with no previous history of any type of cancer, chemotherapy, hormonal or antioxidant therapy, or chronic diseases. Women were excluded if they were current smokers, regular alcohol consumers, antioxidant supplement users, pregnant or lactating, presented hepatic, cardiac, or renal dysfunction, had undergone hormonal therapy, used drugs, or had hypertension, diabetes, and other eventual chronic conditions. Samples from patients treated with NACT were obtained after the completion of chemotherapy treatment and in parallel to samples from patients not treated with NACT and control volunteers in order to be processed under the same conditions. Blood samples were obtained after an overnight fast by venous arm puncture in tubes without anticoagulants. Blood specimens were allowed to clot and centrifuged at 3000× *g* for 10 min at 4 °C to obtain the serum. Serum samples were collected, rapidly frozen in liquid nitrogen, and kept at −80 °C until usage for assays. All procedures performed in studies involving human participants were in accordance with the ethical standards of the institutional and/or national research committee and with the 1964 Helsinki declaration and its later amendments or comparable ethical standards.

### 2.2. Datasets and Pre-Processing

The study subjects were all women of varying ages, as described in Section 2.1. This study aimed to make use of explainable models to accurately predict if a patient had breast cancer.

The dataset included data previously described in [27,28,29,30,31,32,33,34,35] and additional unpublished results. The dataset included electrolytes (sodium, potassium, chloride, calcium, phosphorus, and magnesium), metal ions (iron), albumin, the total protein, and marker proteins (Cerb2, also known as HER2/neu, P53, Ki67, and Bcl-2). It also included enzymes such as adenosine deaminase, CKMB (creatine kinase muscle–brain isoform), amylase, gamma-glutamyl-transferase (GGT), ALAT (alanine aminotransferase) and ASAT (aspartate aminotransferase), acid and alkaline phosphatases, CPK (creatine phosphokinase), and LDH (lactate dehydrogenase). Additionally, it included glucose; non-protein nitrogenous compounds like creatinine, urea, and uric acid; and a lipid profile with the total cholesterol, HDL (high-density lipoprotein), LDL (low-density lipoprotein), and triglycerides. Furthermore, it contained renin–angiotensin system-regulating aminopeptidases (APN, aminopeptidase N; APB, aminopeptidase B; ASAP, aspartyl aminopeptidase; APA, aminopeptidase A; and IRAP, insulin-regulated aminopeptidase). The dataset also contained apolipoproteins A1 and B; hormones including GnRH (gonadotropin-releasing hormone), TSH (thyroid-stimulating hormone), FSH (follicle-stimulating hormone), LH (luteinizing hormone), GH (growth hormone), thyroxine, cortisol, angiotensin, vasopressin, oxytocin, β-endorphin, estradiol, progesterone, and prolactin, along with oxytocinase and pyrrolidone carboxypeptidase hormone-regulatory enzymes; and hormone receptors (ERs, estrogen receptors; PRs, progesterone receptors).

The dataset had several issues, and as such, several pre-processing techniques were applied. The first issue that the dataset had was that some of the attributes in it were specific for patients that had breast cancer and were not applicable to people that belonged to the control class. Consequently, all those attributes were ignored, since they would negatively impact the results of the model due to a bias towards one class if a patient presented any value whatsoever for those attributes. However, no examples were removed from the dataset, as it contained no errors and all data were relevant to the study. As such, it was not necessary to filter the dataset beyond removing those values.

Moreover, many of the attributes had several missing values. In order to fix this issue, the average of the existing values was calculated for each individual class (control or breast cancer), and the result was used to get rid of missing values.

Additionally, since the dataset was quite unbalanced, including roughly twice as many examples of breast cancer patients as healthy people, SMOTE [36] was used as an oversampling technique on the minority class, increasing the amount of examples it included by 50%. There were still more examples of the majority class, but it made the difference less notable. Though more examples could have been added, it would have caused the accuracy of the results to decrease due to an increase in noise in the dataset.

**Figure 1:**
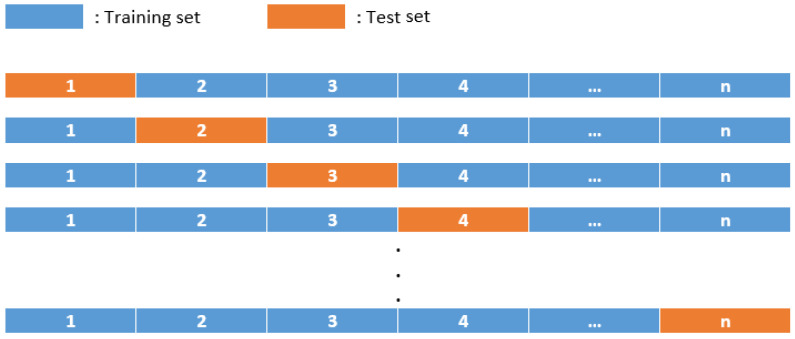
Behavior of the leave-one-out strategy.

Lastly, the strategy used for the training of the models was a leave-one-out strategy, in which each model was trained with all examples in the dataset but one, which was used to test its accuracy. This process was repeated a number of times equal to the amount of examples in the dataset, so that every individual example could be used as a test once, as seen in Figure 1.

### 2.3. Classification Models

In order to solve the classification problem, several models were used in order to compare the results and discover which was most appropriate for the problem at hand. This research focused on models that are both easy to understand and offer highly accurate results.

In order to determine which explainable models to use, the main criterion that was considered for the present study was the output of the model, so that all models provided rules or trees that justified their results and that could be directly linked to the biomarkers that influenced them, thus offering evidence of what biomarkers can provide relevant information that can be easily understood by doctors, potentially aiding them when diagnosing patients. Within these two categories, models that presented highly accurate results (as described in Section 3.1) were selected.

This section includes an explanation of all the models used, as well as the relevant hyperparameters for each model. These hyperparameters were selected due to them providing the most accurate results for each model and were obtained by fine-tuning the models on the training and test datasets.

#### 2.3.1. OneR

One Rule (OneR) [37] is a very simple model that attempts to predict the label of a given dataset by generating one singular rule. It is the natural evolution of the ZeroR model, which simply predicts the majority label. While ZeroR offers no valuable information for predictions, it can be used as a baseline for all the other models, which should achieve higher results.

OneR generates an output, that is, a rule, in two steps. At first, it analyzes all the attributes of the dataset and creates a rule for each of them. Afterwards, it tests every single generated rule to determine which one achieves the highest accuracy. That rule is the one that the model will use for all future predictions.

In spite of its simplicity, OneR can achieve remarkable results on some datasets, particularly those that contain classes that heavily depend on a given attribute or that are relatively simple.

For this specific research, OneR was tested out with a minimum bucket size of 6 for discretizing numerical values, and 4 decimal places were considered.

#### 2.3.2. JRIP

JRIP is the java implementation of the RIPPER [38] algorithm, which generates a set of rules that are then used to predict the labels of later examples. The algorithm creates the ruleset in two stages:

- Building stage: This stage generates rules until the error rate increases beyond a given threshold or no examples remain. A rule is generated by using one attribute, and antecedents are added to it by following a greedy approach [39] until the rule is perfect. Afterwards, rules are incrementally pruned to prevent overfitting.
- Optimization stage: after generating the original ruleset, several variants of each rule are generated and pruned by following the same process as the building stage. One variant is generated by adding more antecedents, and the other is generated from an empty rule. The minimal description length for each original rule and its variants is computed, and the rule that has the lowest description length is selected and added into the final ruleset.

The result is a series of rules that are executed in order and that can accurately describe the behavior of a dataset, which allows for the prediction of items but most importantly allows experts to learn and understand how and why the algorithm labels each example.

In this research, the error threshold was 50%, and two variant rules were generated during the optimization stage.

#### 2.3.3. FURIA

The Fuzzy Unordered Rule Induction Algorithm [40], or FURIA for short, is an algorithm for generating a ruleset that allows for the prediction of labels. It was designed as an extension of JRIP.

The main difference between both algorithms is that the FURIA makes use of fuzzy logic. This implies that rules do not have specific values that need to be met for the antecedent to become relevant; instead, a rule can be triggered with values that are in a certain range with a given confidence factor. It also includes an efficient rule-stretching method to deal with uncovered examples and unordered rulesets.

All of these changes allow the FURIA to improve the results of JRIP in regard to certain problems, particularly for those examples with a large number of attributes or that would require a large ruleset in order to achieve accurate predictions.

As the FURIA is based on JRIP, the hyperparameters used for the FURIA were akin to those used in JRIP. Furthermore, the fuzzy AND operator was defined as the Product T-Norm.

#### 2.3.4. J48

J48 [41] is a decision tree that originated from C4.5 [42]. A J48 tree consists of several nodes which can lead to two or more nodes based on the value of certain attributes of the data. The lowest level nodes, also called leaf nodes, return a given label as the output. Whenever a new example is to be labeled, one must simply follow the nodes of the tree by assessing the values of the attributes of the example.

The tree is built through several iterations of the same algorithm, which takes place in three main stages:

- Stage 1: If all or almost all the elements of a node are of the same class or label, such a node is considered a leaf node, and the algorithm stops for that node.
- Stage 2: Otherwise, for each element in the node, all attributes are considered and tested. The attribute that presents the largest gain in data is selected, and the node is split into several child nodes. Each child node is defined by the threshold values of the selected attribute, so that the items of the parent node are distributed accordingly between all child nodes.
- Stage 3: Once the entire tree has been built by repeating stages 1 and 2, it can be pruned [43] by removing some nodes, thus preventing overfitting and sometimes increasing the accuracy.

J48 has been thoroughly used in many applications and has proven to be able to deal with particular characteristics, lost or missing attribute estimations of the data, and varying attribute costs [44].

The J48 tree used in this research had a confidence factor of 0.25 and a minimum of 2 objects per leaf node. It was also designed not to use the actual value of nodes when it came to splitting them, instead using the value that achieved the highest accuracy. Lastly, during the pruning phase, subtree raising was considered so as to not fully remove them from the resulting tree.

#### 2.3.5. ADTree

The Alternating Decision Tree (ADTree) [45] is a tree-based algorithm. However, unlike decision trees such as J48, the ADTree has two different types of nodes: decision nodes and prediction nodes. Decision nodes work much like nodes in a decision tree would, specifying a singular predicate condition. Prediction nodes, however, have a singular value associated with them.

The ADTree represents each possible label as a numerical value. This way, when an example is examined, it follows the path of the tree, using the decision nodes to decide which prediction nodes are taken into consideration. Once this process is over, the numbers associated with each decision node are added up. The result of this operation is compared to the value of each label. The label with the closest value will be the one predicted. Moreover, this result can double as a confidence factor: if it is very close to the value of the label, it is more likely that the prediction is accurate.

The ADTree has proven to be as robust as boosted decision trees. Although it tends to be more complex than other algorithms that can achieve similar results, several optimizations have been developed to overcome this issue [46].

All of the previously discussed improvements were taken into consideration when designing the ADTree model for this research. Furthermore, the tree was built in 10 boosting iterations, each of which was designed to attempt to expand all possible paths.

#### 2.3.6. Random Forest

The Random Forest model [47] is an ensemble model, and as such, it combines several simpler tree-based models, such as J48, to compute a final result.

What differentiates the Random Forest from other ensemble algorithms is the fact that, for every tree, instead of the full set of attributes, only a fraction of them is considered. Afterwards, the results of each individual tree are combined by using a weighted sum or a majority vote in order to predict the label for a given example.

Since each tree considers different attributes and the information is combined later on, the algorithm becomes more robust, particularly when compared with the individual trees that make up the model. Furthermore, this allows for the partial mitigation of overfitting, as well as any other issues that may arise as a consequence of a high number of attributes.

The Random Forest was generated by using J48 trees, and all previously applicable hyperparameters were still used here. Furthermore, the model was tested by using several different numbers of trees. The best results were achieved by using 20 trees, which were the ones that were considered.

## 3. Results

### 3.1. Metrics

As the accuracy may not be sufficient to determine the quality of a model that deals with the diagnosis of cancer, several metrics were used to compare the models to one another. To understand these metrics, a series of basic definitions upon which they are based is presented:

- True Positive (TP): The amount of patients with breast cancer that were labeled as such.
- True Negative (TN): The amount of patients without breast cancer that were labeled as such.
- False Positive (FP): The amount of patients without breast cancer that were labeled as having breast cancer.
- False Negative (FN): The amount of patients with breast cancer that were labeled as healthy.

With these definitions, we can define the following metrics, which were used to measure the performance of our models:

- Accuracy: (TP+TN)/(TP+FP+TN+FN)
- Precision: TP/(TP+FP)
- Recall: TP/(TP+FN)
- F1 Score: 2×(Precision×Recall)/(Precision+Recall)

### 3.2. General Results

This section includes the results for all models when attempting to detect if a patient had cancer, which can be found in Table 1. Further subsections show the specific results of each model.

#### 3.2.1. OneR

OneR achieved an accuracy of 98.204% by using one singular rule, which implies that the attributes in it could be extremely relevant when attempting to diagnose breast cancer. The rule it generated can be found in Table 2.

#### 3.2.2. JRIP

JRIP generated a ruleset that achieved an accuracy of 98.802% when diagnosing breast cancer. This ruleset can be found in Table 3.

It should be noted that JRIP only generated one rule, which was the same as the one generated by OneR except for some tweaking of specific values, further proving that oxytocin is a key value to consider when diagnosing breast cancer.

#### 3.2.3. FURIA

Unlike the previous algorithms, the FURIA applies fuzzy logic and a confidence factor (CF) to determine how likely a predicted diagnosis is based on the specific values of the data it uses. For each attribute, the entire range of values was divided evenly across five labels: very low, low, medium, high, and very high. Each label was defined by four values; if the example value was between the first and second values or between the third and fourth values, the chosen label was not certain, and the confidence factor was considered. If no other labels were more likely, that label was used. If it was between the second and third values, the label was certain, acting much like those of the previous algorithms. These rules, which achieved an accuracy of 98.802%, are described in Table 4.

The relevant values for the labels are presented hereunder:

- Oxytocin: Very low oxytocin levels were defined by [-Inf, -Inf, 34.8, 36.7]. Very high oxytocin levels were defined by [31.6, 36.7, inf, inf].
- IRAP: Very low levels were defined by [-inf, -inf, 360, 363.859944].

#### 3.2.4. J48

Unlike the previous algorithms, J48 generates a tree which must be followed in order to determine the diagnosis of a given patient. However, such a tree can be easily transformed into an algorithmic set of rules, which can be found in Table 5.

It should be noted that, once more, J48 generated one single rule while achieving an accuracy of 98.204%. However, this time a different attribute was used.

#### 3.2.5. ADTree

The ADTree, much like the previous algorithm, also generates a tree. However, in this case there are two kinds of nodes: decision nodes, to determine which branch of the tree is followed, and prediction nodes, which modify the value of the diagnosis score. Based on the final score, a final diagnosis is provided. The ADTree achieved an accuracy of 98.802%. The results can be represented as a ruleset, which is shown in Table 6. It should be noted that a base value for the score was provided, so that defaultScore =−0.545.

#### 3.2.6. Random Forest

The Random Forest created a total of 25 J48 trees in order to analyze the data and diagnose breast cancer in patients. By using these 25 trees, it achieved an accuracy of 99.401%. By using only the five most relevant trees, the accuracy was over 98%. Those five trees can be found in Table 7, Table 8, Table 9, Table 10, and Table 11, respectively.

It should be noted that the model correctly diagnosed all cancer patients, as shown by the perfect score for its recall.

## 4. Discussion

Though deep learning and AI techniques have been used for the detection of breast cancer in several recent articles in the literature [23,24,48], most of them are fully focused on the prediction of the cancer in itself and less so on the information that can be extracted during the process.

As such, many of the models in the literature oftentimes make use of structures such as autoencoders (AEs) for anomaly detection in images or convolutional neural networks (CNNs) for the analysis of screening mammograms. In [49,50], AEs were used to great effect, achieving an accuracy of roughly 98.5%. In [51], CNNs were used to predict breast cancer using ultrasound images, achieving an accuracy of up to 94%. Similarly, in [52], a CNN model achieved 95.7% accuracy on mammography images. Lastly, in [53], a Quantum Hybrid CNN achieved results of 97.661%.

Though these models can achieve highly accurate results on their own, they lack explainability, since they are black-box models that offer no reasons as to why they predict a given label. Though in many applications of these models this is not an issue, when it comes to healthcare, it is of the utmost importance that experts make informed decisions based on data that back them up. As such, using explainable and trustworthy methods, even if their results are slightly less accurate than those provided by more advanced models, may be preferable. Several studies have made use of machine learning models, such as the Random Forest, to predict breast cancer: in [54], a perfect result was achieved using the Random Forest to diagnose breast cancer according to cell attributes extracted from images, while in [55], a result of 93% was achieved when predicting whether a patient was at risk of developing breast cancer, but the results were based on data gathered from images as well.

Though many other articles have previously used machine learning techniques to detect and prevent breast cancer in the literature [56,57,58,59,60], their results are based on the analysis of images or the exclusive use of medical parameters such as symptoms, weight, and height, among others. Furthermore, whilst several of the proposed models make use of machine learning to predict breast cancer or its recurrence risk, none of them make use of biomarkers and, while the most relevant factors for predictions are sometimes mentioned, the relation and interactions between these factors and breast cancer or its recurrence are hardly explained. This manuscript not only uses biomarkers as a key indicator to predict breast cancer, but also properly defines the given interactions between different biomarkers and the specific threshold values that could be relevant to researchers attempting to diagnose breast cancer.

As can be seen, most of the literature revolves around the use of mammographies and symptom data and extracting information from them, while clinical biomarkers are seldom used. Regardless, the accuracy provided by all the models in this contribution, particularly the Random Forest, which achieved an accuracy of 99.401%, rivals that of some of the state-of-the-art literature while additionally providing valuable information on biomarkers that can guide researchers when it comes to predicting breast cancer.

The used models also provide another advantage: they are easy to understand once generated, and as long as the data that were used to train them are not biased, their results will also be trustworthy. Once a model has generated a ruleset, all that is needed is to check all the generated rules one by one to determine which one is to be used whilst considering biomarker data from a new patient who is to be diagnosed. This prevents biases and limitations of the models when interpreting the results of the models if they have been properly trained.

Nevertheless, these models are not without their own limitations: they require large amounts of quality information in order to properly define the interactions between biomarkers and their potential effect on breast cancer. If data for a specific patient lack values for biomarkers, the models may offer the wrong predictions. Furthermore, model training also has to be carefully revised to avoid biases and overfitting, which may cause models to perfectly predict the training set but offer predictions that are lacking when used outside of it. However, as long as these limitations are taken into consideration and fixed by using pre-processing techniques such as balancing the datasets and leave-one-out validation, the models are able to provide valuable information and accurate results.

In this regard, several important parameters related to breast cancer pathogenesis appear to be involved in several models described here. These parameters can be seen in Table 12 and include peptide hormones, their regulatory proteolytic enzymes, steroid hormones, enzyme activities, and the BMI, among others, being the basis for the explainability of the different models. Of these, oxytocin appeared in all the proposed models except J48.

The following sections provide a thorough explanation of these parameters and their relevance when predicting breast cancer according to the literature.

### 4.1. Peptide Hormones

Oxytocin, a neuropeptide hormone known for its roles in reproduction and social behavior, has emerged as a potentially influential agent in breast cancer development and progression. Its role in this disease, however, is characterized by remarkable complexity, manifesting both proliferative and antiproliferative effects depending on an intricate network of factors including the cell type, hormone concentration, duration of treatment, and interactions with the tumor microenvironment [61,62,63,64,65,66,67]. In fact, oxytocin can inhibit breast cancer cell proliferation through the activation of its receptor (OTR), a member of the G protein-coupled receptor family, triggering an intracellular signaling cascade that can lead to apoptosis, cell cycle arrest, and the suppression of pro-tumor pathways such as PI3K/Akt and ERK [68,69,70,71,72,73]. In vivo studies using animal models of breast cancer have corroborated these findings, demonstrating a reduction in the tumor growth and volume following oxytocin administration [63,64,68,73]. On the other hand, the ability of oxytocin to modulate estrogen receptor (ER) expression and function adds another layer of complexity to its interaction with breast cancer [74,75]. ERs, which play a crucial role in breast cell growth and development, are also important therapeutic targets in breast cancer treatment. Thus, oxytocin, through the modulation of ER expression and activity, could influence the response of tumor cells to hormonal therapies [74,76]. The impact of oxytocin on the expression of microRNAs (miRNAs) has also been the subject of research. miRNAs, small non-coding RNA molecules that regulate gene expression, have emerged as important regulators in cancer. Studies have suggested that oxytocin may influence the expression of specific miRNAs, such as miR-195, which are involved in suppressing tumor growth [73,77]. Despite evidence pointing towards a potential anti-tumor effect of oxytocin, several studies have suggested that this peptide hormone, under certain conditions, may promote breast cancer cell proliferation. The stimulation of angiogenesis, the formation of new blood vessels that nourish the tumor, is one of the mechanisms proposed to explain this effect [78]. In the same way, the overexpression of OTRs, observed in some studies, has been associated with an increase in the metastatic potential of triple-negative breast cancer cells [64,79]. The discrepancy in the results obtained in different studies highlights the importance of context in assessing the effects of OT in breast cancer and could also explain the influence of oxytocin levels in the rules generated in the different models.

GnRH also appeared as an explainable hormonal factor in the J48, ADtree, and Random Forest models. This peptide hormone, produced in the hypothalamus, stimulates the release of luteinizing hormone (LH) and follicle-stimulating hormone (FSH) by the pituitary gland, which in turn induces steroid production in the gonads, playing a key role in the regulation of the reproductive system but also enhancing the growth and progression of estrogen-dependent tumors, as occurs with breast cancer [80,81]. In fact, GnRH analogs are used to suppress ovarian function based on the premise that reducing the circulating estrogen levels can inhibit hormone-dependent tumor growth [82]. It has been recently described that, in premenopausal women with hormone receptor-positive (HR+) breast cancer, ovarian function suppression using GnRH analogs such as goserelin and leuprolide has become an important therapeutic strategy [82]. A meta-analysis of 25 randomized clinical trials, involving nearly 15,000 premenopausal women with HR+ breast cancer, also demonstrated that ovarian ablation or ovarian function suppression using GnRH significantly reduced the 15-year risk of recurrence and mortality [82]. Finally, in addition to the role of GnRH in suppressing ovarian function, there has been research on the use of GnRH analogs as vectors for the targeted delivery of chemotherapeutic drugs to tumor cells. Preclinical studies have shown that the conjugation of GnRH analogs to cytotoxic agents, such as doxorubicin, can increase the treatment efficacy and reduce systemic toxicity [83,84].

The ADtree and Random Forest models also included β-endorphin as an explainable parameter. Neurobehavioural stress can influence the growth and progression of various types of cancer, including breast cancer [35,85]. This is due to the ability of psychological factors to alter the functioning of the immune and endocrine systems, which can affect the tumor growth and spread. Indeed, stress relief in cancer patients promotes faster recovery and a sense of well-being in those receiving radiotherapy or chemotherapy [35,85]. Within this context, opioid peptides, and in particular β-endorphin, have emerged as possible mediators of immune alterations that may contribute to tumor development [35]. β-endorphin plays a crucial role in the bidirectional connection between the immune and neuroendocrine systems, which could explain the effects of stress on the immune capacity against cancer [35,86]. Several studies have shown that healthy women have elevated levels of β-endorphin, with these being even higher in postmenopausal women. However, in women with breast cancer, significantly lower levels are observed, with no differences between premenopausal and postmenopausal women [35]. These findings suggest that decreased levels of β-endorphin may be associated with the development of breast cancer. Neoadjuvant chemotherapy, a common treatment for breast cancer, only improves β-endorphin levels in postmenopausal women, without reaching the levels of healthy women. Furthermore, both premenopausal and postmenopausal women treated with neoadjuvant chemotherapy maintain elevated cortisol levels, indicating a persistent stressful situation [35]. Our findings underscore the significance of our explainable artificial intelligence models in elucidating the critical role of β-endorphin modulation in breast cancer patients to the medical and research community. They could not only facilitate the potential therapeutic implications of β-endorphin regulation, thereby informing clinicians and researchers about novel avenues for intervention in breast cancer management, but could also enhance the understanding of the neuroendocrine basis of breast cancer, potentially guiding future research directions and clinical strategies. In fact, Sánchez et al. (2023) have pointed out that the regulation of stress levels by modulation with β-endorphin could be an alternative pharmacological therapy against tumor growth and development.

Vasopressin was other explainable parameter found in the Random Forest model. Also known as antidiuretic hormone, it has been studied in breast cancer since the presence of vasopressin receptors (V1a, V1b, and V2) in breast cancer cell lines, including MCF-7, were demonstrated [87,88]. Although it has traditionally been attributed a proliferative effect, recent research has suggested that its influence in breast cancer may be more complex and dependent on factors such as the dose, receptor subtype, and cellular context. Thus, a recent study by Alkafaas et al. [89] explored the effects of vasopressin on luminal A breast cancer cells, using a concentration of 100 nM. Their results suggested an anti-proliferative and anti-metastatic effect of vasopressin, with increased apoptosis, the increased expression of apoptosis markers such as Bax and Caspase-3, and decreased cell invasion. In addition, the increased expression of the autophagy marker LC3II and decreased Akt protein activation were found, suggesting the involvement of these pathways in the response to AVP. Garona et al. [90,91] investigated the desmopressin analog [V4Q5]dDAVP, a selective V2 receptor agonist, in preclinical models of breast cancer. They found that this analog inhibited tumor growth, angiogenesis, and metastasis in in vitro and in vivo models. Their findings in MDA-MB-231 xenografts showed reduced tumor growth and angiogenesis with its administration, while in immunocompetent mice with F3II mammary tumors, the complete inhibition of metastatic progression was observed.

### 4.2. Peptide Hormone-Regulating Proteolytic Enzymes

Apart from the previously described peptide hormones, our FURIA, ADTree, and Random Forest models also included the peptide hormone-regulating proteolytic enzymes insulin-regulated aminopeptidase (IRAP) and aminopeptidase B (APB). IRAP, also known as oxytocinase, is a zinc metallopeptidase with important regulatory functions. IRAP is the only known enzyme that cleaves oxytocin and vasopressin in vivo [92,93], two important peptide hormones also included in our proposed models. In addition to its role in hormone regulation, IRAP is also the high-affinity binding site for angiotensin IV (AngIV) type 4 (AT4) receptor ligands [94,95] and is linked to insulin-dependent glucose transporters via the translocation of glucose transporter type 4 (GLUT4) [96]. Previous studies have shown an association between the IRAP activity and the number and size of mammary tumors in an animal model of breast cancer [97,98]. A highly significant increase in IRAP activity has also been found in breast cancer tissue from female patients [99]. Another study by Ramírez-Expósito et al. [31] analyzed the IRAP activity in premenopausal and postmenopausal women with breast cancer, whether or not they had been treated with neoadjuvant chemotherapy. The results showed significant changes in the specific activity of circulating IRAP, with variations dependent on the hormonal status and chemotherapy administration. Thus, IRAP is involved in mechanisms related not only to the regulation of oxytocin and/or vasopressin, but also to the local mammary renin–angiotensin system (RAS) through AngIV and its role in glucose transport through the IRAP/GLUT4 system. The local RAS has been implicated in several of the hallmarks of cancer, including the processes of tumor cell survival, proliferation, and dissemination. The best known RAS signaling is mediated by angiotensin II (AngII) through its type 1 receptor (AT1), which increases cell proliferation and modulates vascular cell growth during angiogenesis [100]. The study by Ramírez-Expósito et al. [31,101] suggested an increase in the circulating levels of AngIV, a substrate of IRAP. AT4 receptor ligands dose-dependently inhibit the catalytic activity of IRAP [102]. Therefore, the changes in the IRAP-specific activity observed in women with breast cancer may be related to local RAS dysregulation. Additionally, mammalian cells use glucose as a major source of energy production. The presence of GLUT4 has been demonstrated in insulin-independent tissues, such as the mammary gland, and also in breast cancer cells, where it plays a role in cell cycle progression [103,104]. IRAP and GLUT4 colocalize in specialized vesicles that translocate to the cell membrane in response to stimuli such as insulin. GLUT4 inhibition in breast cancer cells has been shown to inhibit cell proliferation and decrease cell viability [105]. The changes in IRAP activity observed in [31] could affect the availability of IRAP and, consequently, the translocation of GLUT4 to the cell membrane, which would influence glucose metabolism in tumor cells.

Aminopeptidase B (APB) has also been studied in relation to breast cancer because of its role in the RAS [106]. Thus, APB activity has shown significant patterns of alteration [107]. Martinez et al. [99] found that the membrane-bound APB activity was significantly elevated in neoplastic tissue compared to unaffected tissue, suggesting that APB may play an active role in the tumor microenvironment. Furthermore, in a study by Carrera et al. [108], a decrease in soluble APB was observed in mammary tissue from rats with N-methyl-nitrosourea (NMU)-induced tumors, suggesting that the subcellular location of this enzyme could be an important factor in its influence on a tumor. APB is an enzyme involved in the conversion of angiotensin III (AngIII) to AngIV [108]. Despite the important role of Ang II in cell proliferation, angiogenesis, and tumor growth [106], the metabolism of Ang II, via RAS regulatory enzymes such as APB, is thought to affect tumor progression. Ruíz-Sanjuan et al. [106] suggested that elevated levels of APB may indicate an increased conversion of AngIII to Ang IV, a peptide that binds to AT4 receptors and is also associated with IRAP activity, as previously described. In a two-year follow-up study, Ramirez-Exposito et al. [28] found that APB levels remained significantly unchanged in women with breast cancer. However, a small increase in the APB levels was observed in premenopausal women treated with chemotherapy but was not sustained afterwards. These data suggest that the initial changes in APB activity, associated with tumor development or the effect of chemotherapy, are largely stable over time. Therefore, APB may influence breast cancer development through its role in angiotensin regulation, which may alter cell proliferation and angiogenesis in the tumor microenvironment.

### 4.3. Progesterone

The steroid hormone progesterone was another explainable variable proposed by the Random Forest model. The relationship between progesterone and breast cancer is complex and has been the subject of numerous studies for decades, generating controversy as to whether progesterone is a stimulating or inhibitory factor in the development of this disease [109,110]. Initially, the presence of progesterone receptors (PRs) was recognized as a marker of a response to endocrine therapies and as an indicator of the presence of functional estrogen receptors [109,110]. However, subsequent research has revealed that the action of progesterone can vary depending on factors such as the dose, the type of progestin used, the presence or absence of estrogens, and the clinical context [110,111]. Progesterone exerts its effects through its progesterone receptors (PRs), which are transcription factors that regulate various physiological processes in mammary cells [110]. PRs are found in two isoforms, A and B, which differ in their structure and function [112,113]. These isoforms can operate through different signaling pathways in target cells [112,113]. The activity of PRs is also modulated by the binding of cofactors and by post-translational modifications, such as phosphorylation [113,114]. Progesterone plays a crucial role in the regulation of the cell cycle and proliferation in mammary tissue [111]. It has been observed that progesterone can induce cell proliferation through the activation of the RANKL and WNT4 pathways [110,111,115,116,117,118,119]. In addition, it has been proposed that progesterone can influence the expansion of cancer stem cells, which could contribute to tumor progression [110,115,118,119]. However, there is also evidence that progesterone can have anti-proliferative effects in certain circumstances [120]. The interaction between progesterone and estrogen is fundamental in the context of breast cancer. PRs are target genes regulated by estrogen receptors (ERs) [110,116]. PRs can modulate ER activity, which can have both agonist and antagonist effects [121]. Some studies have suggested that the presence of PRs in ER+ breast tumors is associated with a better response to endocrine therapy [122]. However, the loss of PRs in these tumors has been linked to resistance to treatments such as tamoxifen [123,124,125]. The relationship between endogenous progesterone and the breast cancer risk is not entirely clear [111]. Some studies suggest that elevated levels of progesterone during the luteal phase of the menstrual cycle may increase the risk of breast cancer, especially in women carrying BRCA mutations [111]. It has also been proposed that cumulative exposure to progesterone throughout a woman’s reproductive life could influence the risk [111]. Therefore, progesterone exerts a complex role in the development of breast cancer, with actions that can be both promoting and inhibitory, depending on the cellular and molecular context [110,111].

### 4.4. Other Enzyme Activities

The ADTree model included the 5′-nucleotidase activity as an explainable variable, whereas the Random Forest included the cholinesterase activity as an explainable variable. Ecto-5′-nucleotidase (CD73) is emerging as an enzyme of great interest due to its involvement in the regulation of the tumor microenvironment and its influence on disease progression [126,127,128,129]. This enzyme, located on the cell surface, catalyzes the hydrolysis of 5′-AMP to adenosine and inorganic phosphate [128,130]. Adenosine, the product of this reaction, acts as a potent immunosuppressor in the tumor microenvironment, promoting immune evasion and tumor growth [127,129,131,132]. Its presence in breast cancer is correlated with a worse prognosis, a higher risk of metastasis, and resistance to chemotherapy [127,133]. In particular, in TNBC, a high expression of CD73 has been observed, contributing to treatment resistance and tumor progression [127,134]. Also, CD73 participates in the promotion of tumor angiogenesis, tumor growth, and metastasis [135,136]. On the other hand, some studies have suggested the involvement of cholinesterases in mammary tumorigenesis. In fact, Ruiz-Espejo et al. [137] found that acetylcholinesterase activity doubled in breast tumor tissue compared to in normal tissue. Additionally, the glycosylation of acetylcholinesterase was affected in breast cancer [137]. Bernardi et al. [138] observed a high frequency of amplifications in the gene encoding this enzyme in sporadic breast tumors, which could be responsible for its increased expression. Furthermore, the promoter of the acetylcholinesterase gene contains binding sites for estrogen receptors [139], and the abundance of estrogen receptors in mammary tissue also suggests that these could be involved in regulating acetylcholinesterase expression. Other factors, such as the stimulation of muscarinic receptors, can induce the synthesis of transcription factors that activate the transcription of the acetylcholinesterase gene [140]. Additionally, stimulation by cytokines like interleukin-1β (IL-1β), which is actively synthesized in breast cancer [141], could increase the expression of acetylcholinesterase mRNA and, consequently, its enzymatic activity [142]. Therefore, tumor-specific genetic and biological alterations could influence acetylcholinesterase activity, or its alteration may be a consequence of neoplastic transformations that contribute to the maintenance of the tumor process [138].

### 4.5. Iron

The Random Forest model also proposed serum-circulating iron as an explainable variable for breast cancer diagnosis. Iron is an essential micronutrient for the human body, playing a crucial role in various physiological processes such as oxygen transport, immune function, and energy production [143]. However, excess iron can generate oxidative stress and promote the activation of oncogenes, making it a potentially implicated factor in cancer development [144,145]. Previous epidemiological studies have yielded conflicting results on the association between the serum iron status and breast cancer risk [146]. Some studies have reported a positive association, while others have found no significant relationship [147,148,149,150,151,152]. This inconsistency in results may be attributed to limitations inherent to observational studies, such as the presence of confounding factors and the possibility of reverse causality [153]. To address these limitations, Hou et al. [146] conducted a two-sample Mendelian randomization study to explore the causal relationship between the serum iron status and breast cancer risk. This method used genetic variants as instruments to estimate the causal effect of exposure on an outcome, minimizing bias from confounding factors [154,155]. The results of this study suggested that serum transferrin levels might be positively associated with the risk of estrogen receptor-negative (ER-negative) breast cancer, although no significant effect was observed on the risk of breast cancer in general or its ER-positive subtypes [146]. In another study, Hua et al. [156] retrospectively analyzed the prognostic value of the serum iron level in women with early-stage triple-negative breast cancer (TNBC). They divided patients into two groups based on a cut-off point for the serum iron level and found that those with lower iron levels had significantly longer disease-free survival and overall survival compared to patients with higher levels. These findings suggest that the serum iron level could be a potential prognostic biomarker in patients with early-stage TNBC. It is important to note that most studies assess the iron status in the body by measuring the circulating levels of iron-binding proteins, such as transferrin and ferritin [157,158]. However, the direct measurement of serum iron might provide a more accurate assessment of the iron status in the body [150,151,152,159].

### 4.6. Body Mass Index

The BMI was another of the explainable variables proposed by the Random Forest model. One of the primary mechanisms by which the BMI influences breast cancer outcomes is through its impact on the mammographic imaging quality. Increased adipose tissue in the breast can significantly affect the sensitivity and specificity of mammography, potentially leading to delayed or missed diagnoses. Calvo et al. [160] have described significant differences between the BMI and mammographic density, with a higher BMI in patients with type A density (“fatty breast”) and a lower BMI in patients with type D density (“dense breast”). These differences were more relevant in premenopausal women than in postmenopausal women, although no association was found between the mammographic density and the molecular subtype of cancer [161]. On the other hand, Cao et al. [162] found a positive association between the BMI and breast cancer risk. Women with obesity (BMI >= 28 kg/m^2^) had a 2.09-fold higher risk of developing breast cancer compared to women with a normal weight (BMI of 18.5–23.9 kg/m^2^). A family history of cancer also increased the risk of breast cancer, and an additive interaction was observed between the BMI and family history. In other study, Jiang et al. [163] analyzed the association between the BMI and breast cancer risk in a large cohort of Chinese women, confirming the association with an increased risk of breast cancer, especially in postmenopausal women. Wada et al. [164] also found that in postmenopausal women, a high BMI was significantly associated with an increased risk of breast cancer, but no significant association was observed in premenopausal women. Cubelos-Fernand et al. [165] used the so-called “Clínica Universidad de Navarra Body Fatness Estimator (CUN-BAE)” to assess the relationship between body fat and postmenopausal breast cancer. An increased risk of breast cancer was also observed in women with a higher BMI and CUN-BAE score, with covariates such as the age at recruitment, age at menarche, number of children, and breastfeeding duration also appearing in the multivariate model. Holm et al. [166] investigated the relationship between C-reactive protein (CRP) quartiles and the disease-free survival and overall survival in patients with stage I-III breast cancer. A high CRP level was found to be associated with worse survival in all BMI groups, but the association was more pronounced in overweight and obese women. Jiang et al. [163] focused their study on the relationship between the BMI and the incidence of chronic arm lymphedema (BCRL) after breast cancer surgery. Patients with a BMI >= 30 kg/m^2^ had a significantly higher rate of BCRL at 1, 2, and 3 years compared with patients with a BMI < 25 kg/m^2^. Ogiya et al. [167] also investigated the incidence of lymphedema after breast cancer surgery and its relationship with the BMI. A BMI >= 25 kg/m^2^ was found to be significantly associated with an increased risk of lymphedema at 1 year after surgery. Quartuccio et al. [168] found that the BMI appears to correlate with an increased recurrence rate, especially in the breast, and increased glucose uptake in postmenopausal patients with recurrent breast cancer. Therefore, a complex relationship also exists between the BMI and breast cancer, with a high BMI, especially in postmenopausal women, increasing the risk of breast cancer development, an important variable that must also be taken into account [169].

### 4.7. Total Protein

Very few studies have investigated the relationship between the total serum protein levels and breast cancer. One study revealed that breast cancer patients had significantly higher total serum protein levels compared to the control group [170]. This finding is in agreement with previous studies that have explored the assessment of the total serum protein in patients with brain tumors [171] and with significant increases in the serum protein levels in patients with lung cancer [172]. The increase in the total serum protein concentration may be due to the fact that the total serum protein is composed of albumin and other proteins, collectively called globulins. It is known that the serum albumin concentration can change under oxidative stress, such as that associated with cancer [173]. Furthermore, as plasma circulates through tissues, it picks up proteins released due to physiological events such as tissue remodeling, trauma, and cell death, leading to an increase in the total serum protein.

## 5. Conclusions

This research provides a solid foundation for future clinical research and practical applications, directly challenging conventional approaches to breast cancer diagnosis. The use of XAI models and the identification of relevant serum biomarkers offer a new perspective for early detection, understanding pathogenesis, and developing more effective and personalized therapies. The results not only enrich the scientific knowledge but also open a wide array of possibilities for transforming breast cancer management. They include new approaches to early detection, proposing an alternative to traditional imaging techniques, such as mammography, by focusing on the analysis of serum biomarkers and providing new targets for research and the development of more precise and less invasive diagnostic tools.

Secondly, they provide a deeper understanding of pathogenesis due to the ability of XAI models to generate rules and decision trees that allow researchers to better understand the underlying biological mechanisms in breast cancer development. For example, the identification of oxytocin as a primary predictive factor across several models suggests that this hormone plays a more complex and multifaceted role in pathogenesis than previously thought, which could impact the development of targeted therapies. The detection of complex interactions among hormonal, enzymatic, and metabolic factors opens new avenues for understanding the molecular alterations that drive the disease.

Thirdly, this study lays the groundwork for the design of more individualized treatments by providing information on the specific biomarkers relevant to a patient’s breast cancer risk, an approach that could be especially valuable in managing breast cancers with high heterogeneity and resistance to conventional therapies. Ultimately, this research proposes a transformation in breast cancer management by promoting a more integrated and personalized approach, based on a deeper understanding of the disease and the use of more precise diagnostic tools. The adoption of XAI models could lead to earlier detection, which translates to better survival rates and a reduction in the use of more invasive and costly treatments. Moreover, the models’ capacity to generate explainable information promotes greater confidence in the diagnostic process.

Lastly, the practical challenges of implementing these models in real-world clinical settings should be addressed. All of the used models are highly efficient and simple to implement and can be run even on low-end computers. For these models to be used in a real-world clinical setting, all that would be required would be to store the trained models on a computer, gather all relevant biomarker data from a given patient who has to be diagnosed, and follow the rules generated by a given model. Since several models have been proposed, the data could be run through any of them. This additionally prevent biases and limitations: if all models predict that the patient is likely to develop breast cancer, the chance of a misdiagnosis severely decreases.

## Figures and Tables

**Table 1:** Results for each model when diagnosing a patient.

	Accuracy (%)	Precision	Recall	F1
OneR	98.204 ± 0.134	0.991	0.982	0.987
JRIP	98.802 ± 0.110	0.991	0.991	0.991
FURIA	98.802 ± 0.111	0.991	0.991	0.991
J48	98.204± 0.134	0.991	0.982	0.987
ADTree	98.802 ± 0.110	0.991	0.991	0.991
Random Forest	99.401 ± 0.103	0.991	1.000	0.996

**Table 2:** Rule generated by OneR.

				Precision	Recall
IF	Oxytocin < 35.75	THEN	diagnosis = BreastCancer	0.991	0.982

**Table 3:** Rule generated by JRIP.

				Precision	Recall
IF	Oxytocin < 36.7	THEN	diagnosis = BreastCancer	0.991	0.991

**Table 4:** Rules generated by FURIA.

				Precision	Recall
IF	Oxytocin IS very low	THEN	diagnosis = BreastCancer (CF = 0.99)	0.991	0.991
IF	Oxytocin IS very high AND IRAP IS very low	THEN	diagnosis = Control (CF = 0.98)	0.982	0.982

**Table 5:** Rules generated by J48.

				Precision	Recall
IF	GnRH < 17.5	THEN	diagnosis = BreastCancer	0.991	0.982

**Table 6:** Rules generated by ADTree.

IF	oxytocin < 35.75	THEN	score = score − 2.258
IF	oxytocin >= 35.75	THEN	score = score + 2.012
IF	oxytocin < 85.9	THEN	score = score − 1.151
IF	oxytocin >= 85.9	THEN	score = score + 1.185
IF	APB < 133.396	THEN	score = score + 0.684
IF	APB >= 133.396	THEN	score = score − 0.711
IF	β-Endorphin < 133.396	THEN	score = score − 0.742
**IF**	β-Endorphin >= 133.396	THEN	score = score + 0.858
IF	GnRH < 20.6	THEN	score = score − 0.303
IF	GnRH >= 20.6	THEN	score = score + 0.378
IF	5′-Nucleotidase < 1.45	THEN	score + 0.26
IF	5′-Nucleotidase >= 1.45	THEN	score = score − 0.224
IF	APB < 114.584	THEN	score = score − 0.179
IF	APB >= 114.584	THEN	score = score − 0.177
IF	score < 0	THEN	diagnosis = BreastCancer
ELSE	diagnosis = Control		

**Table 7:** First tree generated by Random Forest.

				Precision	Recall
IF	Vasopressin < 2.68 AND β-Endorphin < 23.3 AND cholinesterase < 7186	THEN	diagnosis = BreastCancer	1.000	0.223
IF	Vasopressin < 2.68 AND β-Endorphin < 23.3 AND cholinesterase >= 7186	THEN	diagnosis = control	1.000	0.055
IF	Vasopressin < 2.68 AND β-Endorphin >= 23.3	THEN	diagnosis = control	0.975	0.710
IF	Vasopressin >= 2.68 AND GnRH < 22.48	THEN	diagnosis = BreastCancer	0.989	0.830
IF	Vasopressin >= 2.68 AND GnRH >= 22.48	THEN	diagnosis = Control	1.000	0.127

**Table 8:** Second tree generated by Random Forest.

				Precision	Recall
IF	GnRH < 20.6 AND Cholinesterase < 8342.5	THEN	diagnosis = BreastCancer	0.991	1.000
IF	GnRH < 20.6 AND Cholinesterase >= 8342.5	THEN	diagnosis = Control	1.000	0.019
IF	GnRH >= 20.6	THEN	diagnosis = Control	0.981	0.964

**Table 9:** Third tree generated by Random Forest.

				Precision	Recall
IF	β-Endorphin < 32.55 AND Protein < 6.79	THEN	diagnosis = BreastCancer	0.984	0.580
IF	β-Endorphin < 32.55 AND Protein >= 6.79 AND Progesterone < 2.15 AND BMI < 28.71 AND oxytocin < 104.24	THEN	diagnosis = BreastCancer	1.000	0.054
IF	β-Endorphin < 32.55 AND Protein >= 6.79 AND Progesterone < 2.15 AND BMI < 28.71 AND oxytocin >= 104.24	THEN	diagnosis = Control	1.000	0.109
IF	β-Endorphin < 32.55 AND Protein >= 6.79 AND Progesterone < 2.15 AND BMI >= 28.71	THEN	diagnosis = BreastCancer	1.000	0.348
IF	β-Endorphin < 32.55 AND Protein >= 6.79 AND Progesterone >= 2.15	THEN	diagnosis = Control	1.000	0.145
IF	β-Endorphin >= 32.55	THEN	diagnosis = Control	0.980	0.890

**Table 10:** Fourth tree generated by Random Forest.

				Precision	Recall
IF	Cholinesterase < 5174	THEN	diagnosis = BreastCancer	0.987	0.688
IF	Cholinesterase >= 5174 AND oxytocin < 35.75	THEN	diagnosis = BreastCancer	1.000	0.277
IF	Cholinesterase >= 5174 AND oxytocin >= 35.75	THEN	diagnosis = control	0.982	0.982

**Table 11:** Fifth tree generated by Random Forest.

				Precision	Recall
IF	β-Endorphin < 28.2 AND Iron < 78.87	THEN	diagnosis = BreastCancer	0.987	0.679
IF	β-Endorphin < 28.2 AND Iron >= 78.87 AND IRAP < 224.96	THEN	diagnosis = BreastCancer	1.000	0.036
IF	β-Endorphin < 28.2 AND Iron >= 78.87 AND 224.96 < IRAP < 264.45	THEN	diagnosis = Control	1.000	0.109
IF	β-Endorphin < 28.2 AND Iron >= 78.87 AND IRAP >= 264.45	THEN	diagnosis = BreastCancer	1.000	0.303
IF	β-Endorphin >= 28.2	THEN	diagnosis = Control	0.980	0.909

**Table 12:** Parameters deemed relevant for the prediction of breast cancer for each tested model.

Parameter	OneR	JRIP	FURIA	J48	ADTree	Random Forest
Hormones	Oxytocin	Oxytocin	Oxytocin	GnRH	Oxytocin, GnRH, β-endorphin	Oxytocin, GnRH, β-endorphin, vasopressin, progesterone
Hormone-regulating proteolytic enzymes			IRAP		APB	IRAP
Other enzyme activities					5′-Nucleotidase	Cholinesterase
Metal ions						Iron
Other						Total protein, BMI

IRAP: insulin-regulated aminopeptidase; GnRH: gonadotrophin-releasing hormone; APB: aminopeptidase B; BMI: body mass index.

## Data Availability

The data are unavailable due to privacy or ethical restrictions.

## Abbreviations

The following abbreviations are used in this manuscript:

ADTree	Alternating Decision Tree
AI	Artificial intelligence
ALAT	Alanine aminotransferase
AngII	Angiotensin II
AngIII	Angiotensin III
AngIV	Angiotensin IV
APA	Aminopeptidase A
APB	Aminopeptidase B
APN	Aminopeptidase N
ASAP	Aspartyl aminopeptidase
ASAT	Aspartate aminotransferase
AT1	Angiotensin II type 1 receptor
BCRL	Breast cancer-related lymphedema
CF	Confidence factor
CKMB	Creatine kinase muscle–brain isoform
CNN	Convolutional neural network
CPK	Creatine phosphokinase
CRP	C-reactive protein
CUN-BAE	Clínica Universidad de Navarra Body Fatness Estimator
DCIS	Ductal carcinoma in situ
ER	Estrogen receptor
FN	False Negative
FP	False Positive
FSH	Follicle-stimulating hormone
FURIA	Fuzzy Unordered Rule Induction Algorithm
GGT	Gamma-glutamyl-transferase
GH	Growth hormone
GLUT4	Glucose transporter type 4
GnRH	Gonadotropin-releasing hormone
HDL	High-density lipoprotein
HR+	Hormone receptor-positive
IRAP	Insulin-regulated aminopeptidase
JRIP	Java implementation of the RIPPER algorithm
LDH	Lactate dehydrogenase
LDL	Low-density lipoprotein
LH	Luteinizing hormone
miRNAs	MicroRNAs
MRI	Magnetic resonance imaging
NMU	N-methyl-nitrosourea
OneR	One Rule
OTR	Oxytocin receptor
PR	Progesterone receptor
RANKL	Receptor activator of nuclear factor kappa-B ligand
RAS	Renin–angiotensin system
RNN	Recurrent neural network
TAI	Trustworthy artificial intelligence
TN	True Negative
TNBC	Triple-negative breast cancer
TP	True Positive
TSH	Thyroid-stimulating hormone
WNT4	Wingless-related integration site 4
